# Supplementary material for: LGR signaling mediates muscle-adipose tissue crosstalk and protects against diet-induced insulin resistance
Source: Nat Commun. 2024 Jul 20;15:6126. doi: 10.1038/s41467-024-50468-w (PMC11271308; doi:10.1038/s41467-024-50468-w)
Supplement: Supplementary file 1 — Supplementary Information [file 41467_2024_50468_MOESM1_ESM.pdf]

Supplementary Information

LGR signaling mediates muscle-adipose tissue crosstalk and protects against diet-induced insulin resistance

Olga Kubrak[1,†], Anne F. Joergensen[1, 2,†], Takashi Koyama[1,†], Mette Lassen[1,†], Stanislav Nagy[1], Jacob Hald[2], Gianluca Mazzoni[2], Dennis Madsen[2], Jacob B. Hansen[1], Martin Røssel Larsen[3], Michael J. Texada[1], Jakob L. Hansen[2], Kenneth V. Halberg[1], and Kim Rewitz[1,*]

[†]Equal contribution

[1]Department of Biology, University of Copenhagen, 2100 Copenhagen O, Denmark

[2]Novo Nordisk, Novo Nordisk Park, 2760 Maaløv, Denmark

[3]Department of Biochemistry and Molecular Biology, University of Southern Denmark, 5230 Odense, Denmark

*Correspondence: Kim.Rewitz@bio.ku.dk

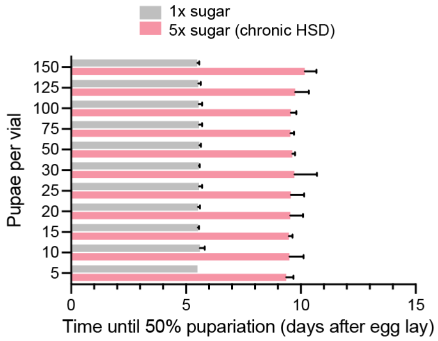

Supplementary Figure 1. Time until 50% pupariation for animals raised on normal or high-sugar diet at 25 °C, at a range of population densities. Statistics: Error bars represent mean and SEM. Time until 50% pupariation was determined via linear interpolation between adjacent data points above and below 50%. No significant differences were observed. Source data are provided as a Source Data file.

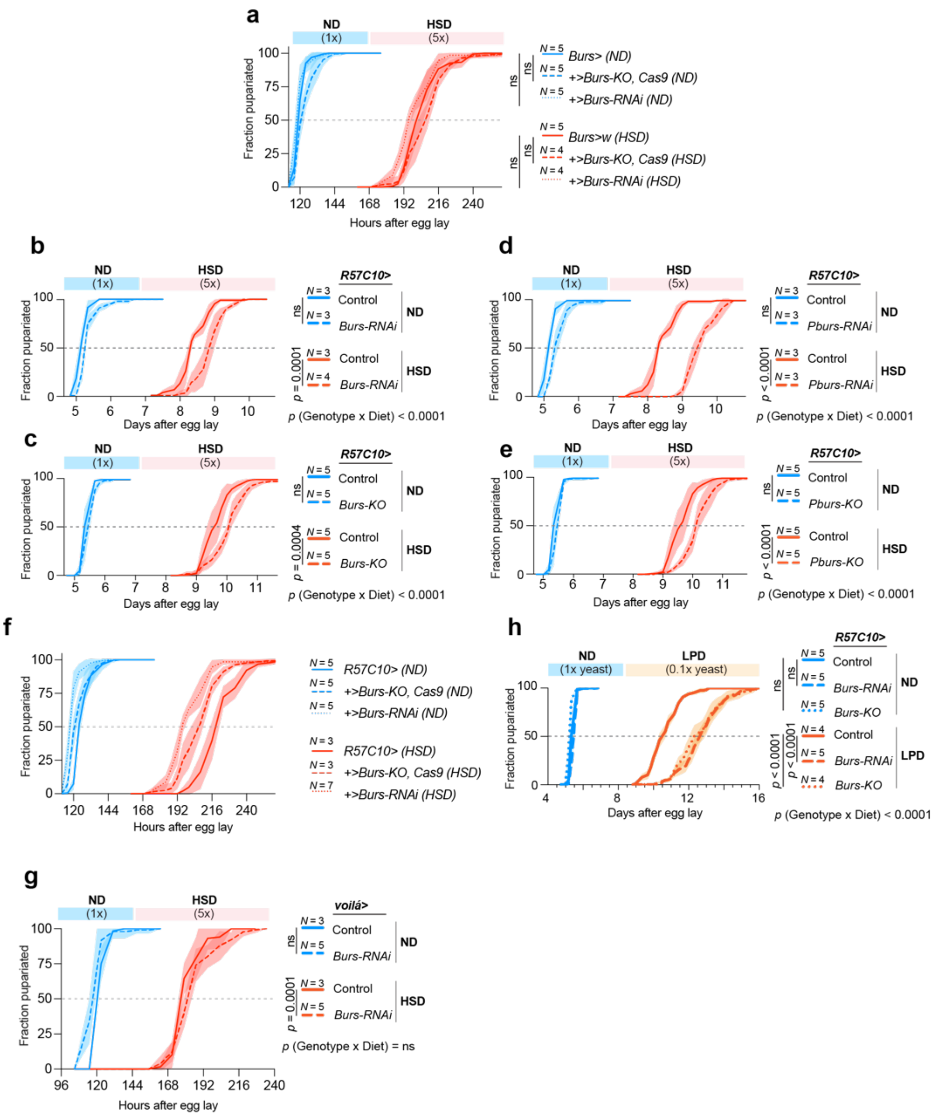

Supplementary Figure 2. (A) Time of pupariation was compared between GAL4 driver controls (*Burs>*) and animals (raised at 29 °C) harboring either *UAS-Burs-RNAi* or *UAS-Burs-KO* constructs (crossed to w^{1118}) in the absence of the GAL4 driver, to exclude any potential effects of the genetic background of the RNAi or CRISPR constructs on the delay phenotypes observed with a high-sugar diet. (B,D) Comparison of the timing of pupariation between controls and animals expressing pan-neuronal knockdown of *Burs* or *Pburs*, raised on normal or high-sugar diet at 29 °C. Experiments measuring pupariation timing in animals lacking *Burs* and *Pburs* were performed concurrently and shared the ND and HSD control, but the data are presented in separate figures (B, D) for clarity. (C,E) Comparison of the timing of pupariation between controls and animals expressing pan-neuronal somatic deletion of *Burs* or *Pburs*, raised on normal or high-sugar diet at 29 °C. As with the knockdowns, the timing experiments for the two knockouts were performed concurrently and shared the ND and HSD control, but data for the two knockouts are presented separately (C, E) to reduce graphical crowding. (F) Timing of pupariation at 29 °C was compared between GAL4 driver controls (*R57C10>*) and animals harboring either *UAS-Burs-RNAi* or *UAS-Cas9, UAS-Burs-KO* constructs (crossed to w^{1118}) in the absence of the GAL4 driver, to exclude any potential effects of the genetic background of the RNAi or CRISPR constructs on the delay phenotypes observed with a high-sugar diet. (G) Pupariation timing was compared between controls and animals expressing RNAi against *Burs* in all entero-endocrine cells using *voilà-GAL4* (*voilà>*), raised at 25 °C on normal or high-sugar diet. (H) Pupariation timing on normal and low-yeast diets for animals (raised at 29 °C) with pan-neuronal knockdown or knockout of *Burs*. Statistics: Shading indicates SEM. ns, not significant (p>0.05). A, B, C, D, E, G, H: one-way ANOVAs with Tukey's correction between 50%-pupariation times for multiple comparisons and two-way ANOVA for interaction. The time of 50% pupariation was determined via linear interpolation between adjacent observations before/below and after/above 50%. Source data are provided as a Source Data file.

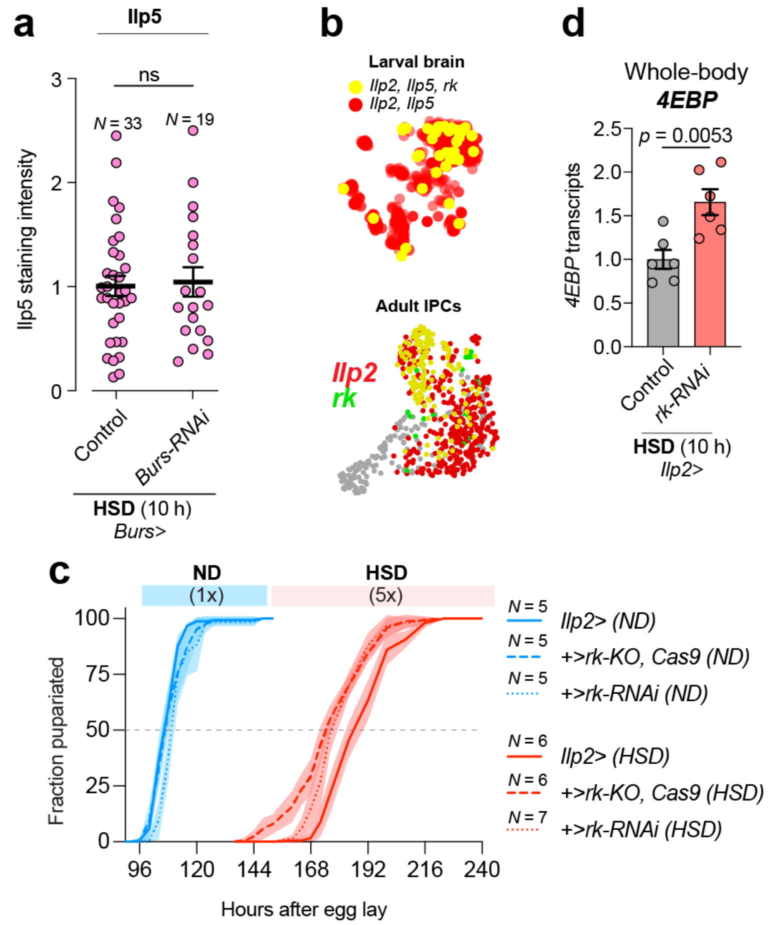

Supplementary Figure 3. (A) Quantification of anti-Ilp5 staining intensity in multiple samples from animals with Burs^{+}-cell *Burs* knockdown. (B) Single-cell transcriptomics data demonstrate overlap between insulin-gene and *rk* expression, consistent with *rickets* expression in the larval and adult IPCs. Top: Data from dissected larval brains[1] analyzed with Trailmaker (Parse Biosciences). Cells expressing both *Ilp2* and *Ilp5* are shown in red, with those also giving detectable *rk* in yellow. Bottom: Data from sorted adult IPCs (data and analysis via FlyCellAtlas[2]). Yellow marks IPCs expressing both *Ilp2* and *rk*. In both cases, the true fraction of *rk*$^{+}$ cells is likely higher than pictured, given the low expression level of GPCRs and the practical limitation of sequencing depth. (C) Time until 50% pupariation at 29 °C was compared between GAL4 driver controls (*Ilp2>*) and animals harboring either *UAS-rk-RNAi* or *UAS-rk-KO+UAS-Cas9* constructs (crossed to w^{1118}) in the absence of the GAL4 driver, to exclude any potential effects of the genetic background of the RNAi or CRISPR constructs on the delay phenotypes observed in animals fed high-sugar diet. (D) Whole-body *4EBP* transcript levels measured by qPCR in controls and animals with *rickets* knockdown in the insulin-producing cells, raised on normal diet for 90 hours and then fed with a 10-hour pulse of normal or high-sugar diet. Statistics: Mean and SEM are represented by error bars in panels A and D and by shading in panel C. ns, not significant (p>0.05). A and D, two-sided unpaired Student's t-test. The time of 50% pupariation was determined via linear interpolation between adjacent observations before/below and after/above 50%. Source data are provided as a Source Data file.

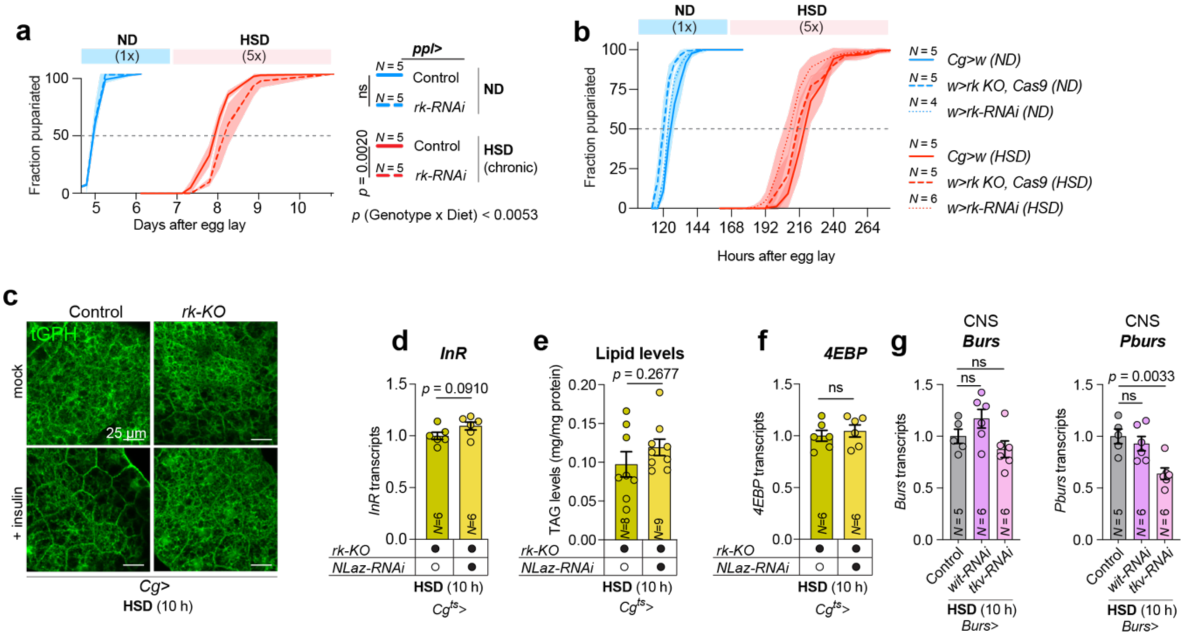

Supplementary Figure 4. (A) Pupariation timing of controls and animals expressing fat-body-specific *rk* knockdown using *ppl-GAL4* (*ppl>*), raised on normal or high-sugar diet at 25 °C. (B) Time until 50% pupariation at 29 °C was compared between GAL4 driver control (*Cg>*) and animals harboring either *UAS-rk-RNAi* or *UAS-rk-KO+UAS-Cas9* constructs (crossed to w^{1118}) in the absence of the GAL4 driver, to exclude any potential effects of the genetic background of the RNAi or CRISPR constructs on the delay phenotypes observed with a high-sugar diet. (C) Representative images of tGPH insulin-indicator responses to exogenously applied human insulin in fat-body explants from controls and animals expressing CRISPR-mediated deletion of the *rickets* locus in the fat body, exposed to high-sugar diet for 10 hours before dissection. Scale bars, 25 microns. (D, F, E) qPCR analysis of *Insulin receptor* (*InR*) and *4E-binding protein* (*4EBP*) transcript levels and triacylglyceride (TAG) levels measured in the whole body of animals with a fat-body-specific deletion of the *rk* locus (*rk-KO*) alone and in combination with *NLaz-RNAi*, after 10 hours on a high-sugar diet (HSD). Expression was driven using the *Cg-GAL4* driver combined with *Tub-GAL80*^{TS} (*Cg*^{TS}*>*) to investigate immediate effects. For inducible fat-body-specific expression, activation coincided with the transfer to HSD (144 hours after egg collection) by shifting animals from 18 °C to 29 °C. (G) Transcript levels of *Burs* (left) and *Pburs* (right) measured by qPCR in dissected CNS samples from controls and animals expressing RNAi against *wit* or *tkv* in the *Burs*-expressing cells, fed high-sugar diet for 10 hours. Statistics: Shaded areas in panels A and B and error bars in other panels represent mean and SEM. ns, not significant (p>0.05). A,D,E,F: two-sided unpaired Student's t-test. G: one-way ANOVAs with Dunnett's multiple-comparisons test. The time of 50% pupariation was determined via linear interpolation between adjacent observations before/below and after/above 50%. Source data are provided as a Source Data file.

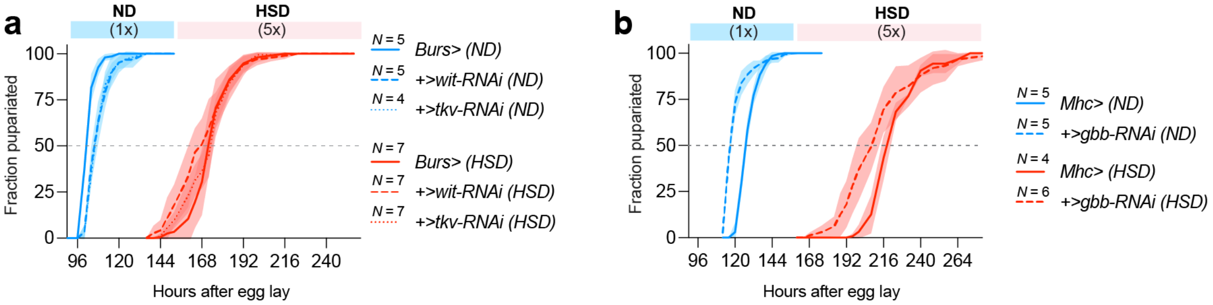

c

Exact p values for Fig. 7f.

	Time (min)																			
	0.5	1	1.5	2	2.5	3	3.5	4	4.5	5	5.5	6	6.5	7	7.5	8	8.5	9	9.5	10
IH vs IL	0.0156	0.0312	0.1094	0.0625	0.0156	0.0156	0.0156	0.0156	0.0156	0.0469	0.0156	0.0156	0.0156	0.0156	0.0156	0.0312	0.0156	0.0156	0.0312	0.0156
IL vs SH	0.8906	0.3750	0.0469	0.0792	0.0469	0.1562	0.1562	0.0625	0.0469	0.0469	0.1562	0.1250	0.1094	0.2969	0.2969	0.4062	0.5781	0.0469	0.0469	0.1562

Supplementary Figure 5. (A, B) Time until 50% pupariation at 29 °C was compared between GAL4 driver controls (*Burs>* or *Mhc>*) and animals harboring *UAS-wit-RNAi, UAS-tkv-RNAi*, or *UAS-gbb-RNAi* constructs (crossed to w^{1118}) in the absence of the GAL4 driver, to exclude any potential effects of the genetic background of the RNAi or CRISPR constructs on the delay phenotypes observed with a high-sugar diet. (C) Exact *p* values for comparisons between treatments at each time point in Fig. 7f using Wilcoxon matched-pairs signed rank test (two-tailed). Statistics: Shaded areas in A and B represent SEM. The time of 50% pupariation was determined via linear interpolation between adjacent observations before/below and after/above 50%. Source data are provided as a Source Data file.

Supplementary Table 1. A summary of genetic evidence from various studies that investigate the association between LGR4 and obesity or type-2 diabetes (T2D) in humans or mice.

Reference	Species	Tissue	LGR4 mutations	LGR4 expression and activity	Obesity and T2D related traits
Wang *et al.*, 2013[3]	Human	N/A	LGR4 A750T	Gain of function	↑Obesity occurrence
Wang *et al.*, 2013[3]	Human	Intra-abdominal/ subcutaneous adipose tissue	N/A	↑ mRNA expression	↑Obesity occurrence
Styrkarsdottir *et al.*, 2013[4]	Human	N/A	*Lgr4 C→T*	Loss of function (premature stop and nonsense-mediated mRNA decay)	•↓ glucose levels<br>•↓ body weight (↓ lean mass)
Li *et al.*, 2019[5]	Human	Blood serum	N/A	↓ protein abundance	• T2D occurrence<br>•↑HbA1c
Zou *et al.*, 2016[6]	Human	N/A	LGR4 A750T	Gain of function	•↑ waist circumference<br>•↑ waist-to-height ratio<br>•↑ waist-to-hip ratio<br>•↑ abdominal visceral fat area<br>•↑ 2-h plasma insulin<br>•↓ Matsuda index
Wang *et al.*, 2013[3]	Mouse	N/A	$Lgr4^{m/m}$	*Lgr4* KO	• ↓ adiposity<br>•resist dietary and leptin mutant-induced obesity<br>•improved glucose metabolism
Luo *et al.*, 2009[7]	Mouse	N/A	$Lgr4^{-/-}$	*Lgr4* KO	•↓ body weight (fetal development)
Sun *et al.*, 2018[8]	Mouse	N/A	$Lgr4^{-/-}$	*Lgr4* KO	•↓ fat mass<br>•↓ white adipogenesis<br>•↑ brown adipogenesis

Supplementary references

1 Brunet Avalos, C., Maier, G. L., Bruggmann, R. & Sprecher, S. G. Single cell transcriptome atlas of the Drosophila larval brain. *Elife* **8** (2019). https://doi.org:10.7554/eLife.50354

2 Li, H. *et al.* Fly Cell Atlas: A single-nucleus transcriptomic atlas of the adult fruit fly. *Science* **375**, eabk2432 (2022). https://doi.org:10.1126/science.abk2432

3 Wang, J. *et al.* Ablation of LGR4 promotes energy expenditure by driving white-to-brown fat switch. *Nat Cell Biol* **15**, 1455-1463 (2013). https://doi.org:10.1038/ncb2867

4 Styrkarsdottir, U. *et al.* Nonsense mutation in the LGR4 gene is associated with several human diseases and other traits. *Nature* **497**, 517-520 (2013). https://doi.org:10.1038/nature12124

5 Li, B. *et al.* Type 2 diabetes with hypertensive patients results in changes to features of adipocytokines: Leptin, Irisin, LGR4, and Sfrp5. *Clin Exp Hypertens* **41**, 645-650 (2019). https://doi.org:10.1080/10641963.2018.1529779

6 Zou, Y. *et al.* Association of a gain-of-function variant in LGR4 with central obesity. *Obesity (Silver Spring)* **25**, 252-260 (2017). https://doi.org:10.1002/oby.21704

7 Luo, J. *et al.* Regulation of bone formation and remodeling by G-protein-coupled receptor 48. *Development* **136**, 2747-2756 (2009). https://doi.org:10.1242/dev.033571

8 Sun, P. *et al.* Loss of Lgr4 inhibits differentiation, migration and apoptosis, and promotes proliferation in bone mesenchymal stem cells. *J Cell Physiol* **234**, 10855-10867 (2019). https://doi.org:10.1002/jcp.27927
