# Supplementary material for: LGR signaling mediates muscle-adipose tissue crosstalk and protects against diet-induced insulin resistance
Source: Nat Commun. 2024 Jul 20;15:6126. doi: 10.1038/s41467-024-50468-w (PMC11271308; doi:10.1038/s41467-024-50468-w)
Supplement: Supplementary file 3 — Description of Additional Supplementary Files [file 41467_2024_50468_MOESM3_ESM.docx]

**Description of Additional Supplementary Files**

**Supplementary Data 1:** In-vivo RNAi screening data; 2,256 genes were tested in a Drosophila model of diet-induced obesity for sugar-induced developmental delays or impaired survival.

**Supplementary Data 2:** Quantitative phosphoproteomics data from mouse adipocytes. Responses to insulin stimulation in controls and adipocytes with Lgr4 knockdown.

**Supplementary Data 3:** Reactome pathway analysis of phosphoproteomics data from mouse adipocytes after insulin stimulation, comparing controls and adipocytes with Lgr4 knockdown.

**Supplementary Data 4:** Genotypes of animals used for each figure.

**Supplementary Data 5:** Oligos and DNA sequences for gRNA constructs, CRISPR knock-in, and qPCR
