# Supplementary material for: LGR signaling mediates muscle-adipose tissue crosstalk and protects against diet-induced insulin resistance
Source: Nat Commun. 2024 Jul 20;15:6126. doi: 10.1038/s41467-024-50468-w (PMC11271308; doi:10.1038/s41467-024-50468-w)
Supplement: Supplementary file 8 — Supplementary Data 5 [file 41467_2024_50468_MOESM8_ESM.docx]

**Supplementary Data 5.**

Oligos for gRNA constructs

*Burs-KO-F:* CGGCCCGGGTTCGATTCCCGGCCGATGCAGATTCCCTCGTTCGCCTGCGGTTTCAGAGCTATGCTGGAAAC

*Burs-KO-R:* ATTTTAACTTGCTATTTCTAGCTCTAAAACCAGGAAATTGCCGGCTATTCTGCACCAGCCGGGAATCGAACC

*Pburs-KO-F*: CGGCCCGGGTTCGATTCCCGGCCGATGCAGAAGCATGGATATACGTCTTGTTTCAGAGCTATGCTGGAAAC

*Pburs-KO-R:* ATTTTAACTTGCTATTTCTAGCTCTAAAACCCTTGATCAAGTGGATCTCCTGCACCAGCCGGGAATCGAACC

*rk-KO-F:* CGGCCCGGGTTCGATTCCCGGCCGATGCAGTTGCTACCTGAAGATGTACGTTTCAGAGCTATGCTGGAAAC

*rk-KO-R:* ATTTTAACTTGCTATTTCTAGCTCTAAAACTTCACCTGAACAAGAGGCTCTGCACCAGCCGGGAATCGAACC

FLAG::Bursicon::HA knock-in sequence

Homology1 UTR Signal sequence FLAG tag Burs intron Burs intron Burs HA tag UTR homology2

TACGAGGAGAATCCTGACTTGGTTCAGGCAATTTTCGAAATGATTACCGAAAACATTATGGAATTAGCGTATAAGTGAGTTATTTTTCATAATTTGATCATCTGAGAGTGTGGTTTGATTGTTTATTGAACACTTAACCTTGTCGCTTTTCAGCTTCCAGAGTTGGTCGGGACACGGCCGAGGTGATCAATGACATGGCGGAGTACTTCACCACCCGGGAGCAACAGCAGTTGCTCAAGGAATTCTACTACAGCAATCACCAGTTCTTCGGTGAATCGGAAGAGCTGCTCTCCAAGTCCCTGGTCACTGTGGAAAAAAACCTTAACTGGGCGGAAACCCATTTGGAAGGCTTAGTAAAATATCTGGCGGACAGAAATGGTAGCTCCTGCCTGGGAGCCACTTCCTTGGTGGTGGTTCTGCTTTTAGCAGTGGGCAGCATGTTGGCCTGGCCCTGATTAGCCAATAAGTTGTGAGGGAAATAAAGAGATTGCCGTGGTCGTGCGCCGCGGGAGAAATGCCCTTTTCGTGTACGCAATTACTAGGCAAAAAGGTTTGGACGGCCCTTGCTTTATTAGGCCATAATTTACGCTGTCGAGCGGCCATTAGAGCGGCATCCGCTGGCCAAGTTGGTCTATAAAAGCCCGGCGGCTGCAAGTGGCGTTTTCCATTCCACGTGAAAGGACACTCGCAGTCGGGCCGACGAG**ATG**CTGCGCCACCTGCTCCGCCACGAGAACAACAAGGTCTTCGTCCTGATCCTGCTCTACTGCGTCCTGGTCAGCATTCTGAAACTCTGCACGGCAGACTACAAAGACGATGACGACAAGCAGCCGGATAGCTCTGTGGCCGCCACGGATAATGGTATTTGATAAGGCATTTGATAATATCATATTATTCGCTCTAATGGATTCCATTTACCATCCAGATATTACGCATCTTGGCGACGATTGTCAGGTGACGCCCGTCATCCATGTGCTCCAGTATCCTGGATGTGTGCCCAAGCCGATTCCCTCGTTCGCCTGCGTGGGTCGCTGTGCCAGTTATATCCAGGTGGGTGTTGATATACTTGAAATATAAATTATAATTTGATATAATCTGATGACTTTAGGTTTCGGGCAGTAAGATCTGGCAAATGGAGCGTTCCTGCATGTGCTGCCAGGAGTCTGGTGAGCGGGAGGCAGCCGTCTCGCTATTCTGTCCCAAAGTGAAGCCCGGCGAGCGTAAATTCAAGAAGGTCCTGACCAAGGCGCCATT[G🡪A]GAGTGCATGTGTCGGCCATGCACTTCCATTGAGGAGTCTGGCATCATACCACA[G🡪A]GAAATTGCCGGCTATTCGGACGAGGGTCCACTCAACAATCACTTCCG[G🡪C]CGCATTGCTCTGCAATACCCATACGATGTTCCAGATTACGCT**TAG**ATTCCCCCATCAGTTTAGCACTCATACCCATGCCCATTTGCTCATTAAAATAAATTAGAGTTGCCGTTTCTGCTTGTACACAGTGAAAAATGTGCCTCTGAAAGCTCATGCCAGTGAAAGGGAAACATCAGCAGATATATGATTATTTTAAATTGTAATTTATTTCCCAACTATCTCATGTCATTGCTACTAGTTCACTAAATGGTGTGTGTCCTGCTTCACAGGGTATGTATGTAGATGCGTAACTCCAAAGAAAACAAAGATAATCTTTACACTGCAACTGGATCTAACAATAGGAGTTTAACTACGGCATAATGCATATGACTCGCGCCGCCTACAAGTACTCGACGATATCGCATTTGATGGATATATTCGGACAGCTCTGGCCCACGGCACTGCGGCCCCGATAGTTGGGACTCTTCTCGGTGAGGAGTTTCCGGAAGATGGGCAAAATTGCCGTCTCGCCCTCGAGCAGTTCCGTCTCGCTGCTGGCGCCGTAGATGCTGCTATCCTGCTTGCCGCGCTGCCTGCTCCGCTGCTTATCGGGCGCCACATTGGGCGAACTGTGGTGGTGGGCGGCGTGCTGGATGTTAGAGCTCCGGCTGACCTTACGCGGCTGGACGCACGGCTGACTGTTGGGCGGCGGATCTCGTTCCCGTTCGCTGTCCGTCTCGCTCTGGCAAACGGTGCCCTTTTGCGGTTTTGGCCGATGTTTGTTGCGGTTCTTCTCGGAGACGGACCGCTGGATGGGCGAGGTGGTGTTCGGGTCATAGGTCATCCCGTGGCCGCCATGATGT

Oligos for CRISPR knock-in

Burs-gRNA1-F: GTCGTCCACTCAACAATCACTTC

Burs-gRNA1-R: AAACGAAGTGATTGTTGAGTGGA

Burs-gRNA2_F: GTCGGAGTCTGGCATCATACCAC

Burs-gRNA2_R: AAACGTGGTATGATGCCAGACTC

Burs-gRNA3_F: GTCGGTCCTGACCAAGGCGCCAT

Burs-gRNA3_R: AAACATGGCGCCTTGGTCAGGAC

Oligos for qPCR

4EBP-F: TGCCCATGATCACCAGGAAG

4EBP-R: TCGTAGATAAGTTTGGTGCCTCC

Burs-F: CATCCATGTGCTCCAGTATCC

Burs-R: GGCTTCACTTTGGGACAGAA

gbb-F: GCGAGTGCAATTTCCCGCTCAATG

gbb-R: CAGGTTCACATTCTCGTCGTTCAGG

Ilp2-F: CTCAACGAGGTGCTGAGTATG

Ilp2-R: GAGTTATCCTCCTCCTCGAACT

Ilp3-F: AGAGAACTTTGGACCCCGTGAA

Ilp3-R: TGAACCGAACTATCACTCAACAGTCT

Ilp5-F: ATGGACATGCTGAGGGTTG

Ilp5-R: GTGGTGAGATTCGGAGCTATC

NLaz-F: ACGCCAACTACAGTCTCATAGA

NLaz-R: CGAGGGTTGTCCGGTGAATC

Pburs-F: AGGATTGTGCAACAGTCAGG

Pburs-R: AGCAATGGGTTAGAGTGATGA

InR-F: CTCAGCCATACCAGGGACTTT

InR-R: CTCTCCATAACACCGCCATC

Rp49-F: AGTATCTGATGCCCAACATCG

Rp49-R: CAATCTCCTTGCGCTTCTTG
